# Supplementary material for: Pathogenome and Plasmid-Borne Antimicrobial Resistance Phenotypes in a Multidrug-Resistant O111:H8 Shiga Toxin-Producing Escherichia coli Strain
Source: bioRxiv. 2026 Sep 18:2026.09.16.752176. Preprint. [Version 1] doi: 10.64898/2026.09.16.752176 (PMC13596339; doi:10.64898/2026.09.16.752176)
Supplement: Supplementary file 1 [file NIHPP2026.09.16.752176v1-supplement-1.pdf]

bioRxiv preprint doi: https://doi.org/10.64898/2026.09.16.752176; this version posted September 18, 2026. The copyright holder for this preprint (which was not certified by peer review) is the author/funder, who has granted bioRxiv a license to display the preprint in perpetuity. It is made available under a CC-BY 4.0 International license.

Supplemental Figures

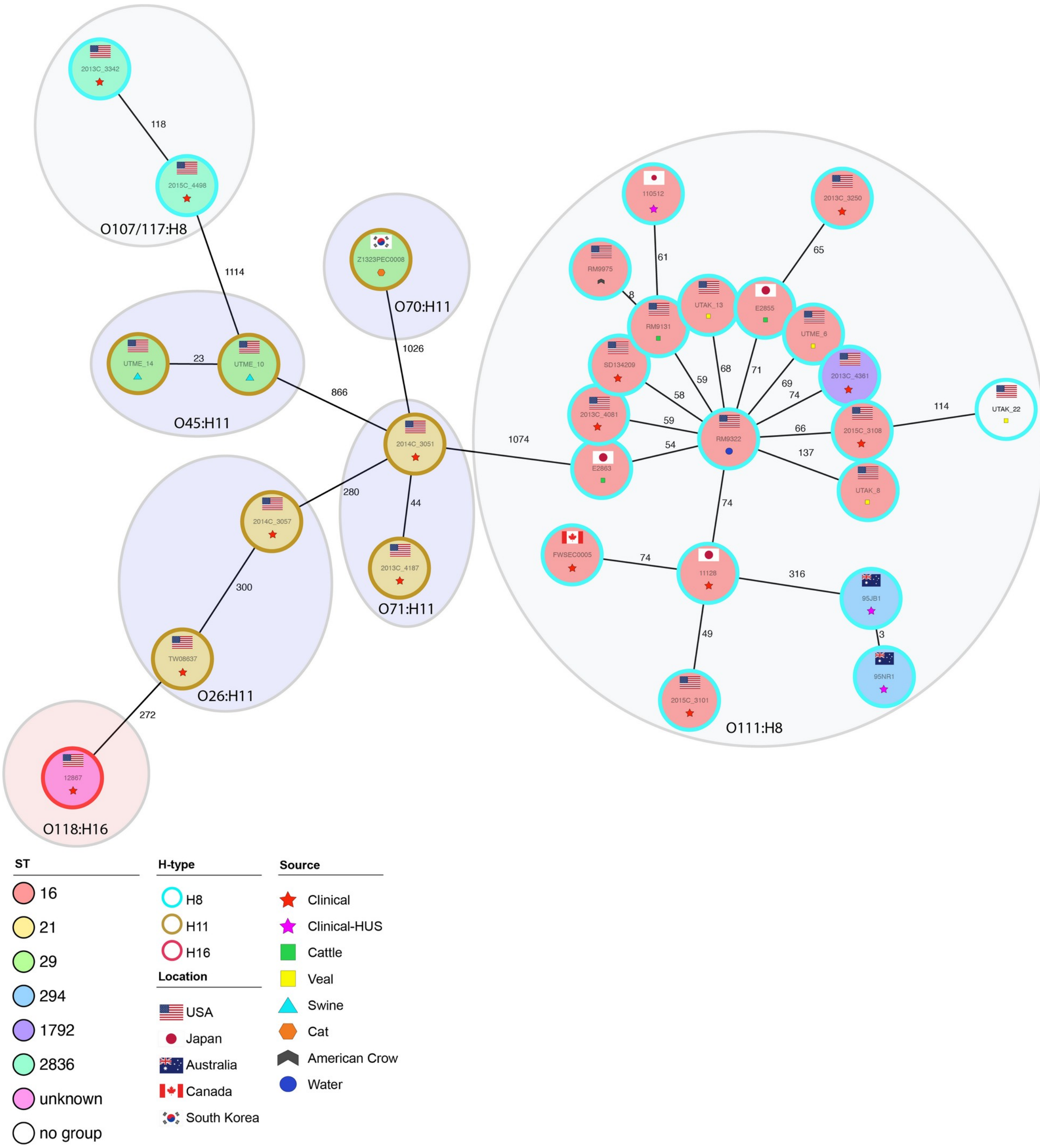

FIG S1. **Core-genome phylogeny of UTAK-22 and related *E. coli* genomes.** The phylogenetic position of UTAK-22 and the 30 most closely related *E. coli* genomes identified by Mash-based similarity analysis was inferred using a cgMLST minimum-spanning tree generated in Ridom SeqSphere+. The shared gene

bioRxiv preprint doi: https://doi.org/10.64898/2026.09.16.752176; this version posted September 18, 2026. The copyright holder for this preprint (which was not certified by peer review) is the author/funder, who has granted bioRxiv a license to display the preprint in perpetuity. It is made available under a CC-BY 4.0 International license.

inventory comprises 4,160 genes, including 3,190 core and 970 accessory loci. Allelic distances between nodes are indicated on the connecting lines. Node color and ring annotations denote sequence type, H-antigen type, source, and geographic origin as indicated in the legend.

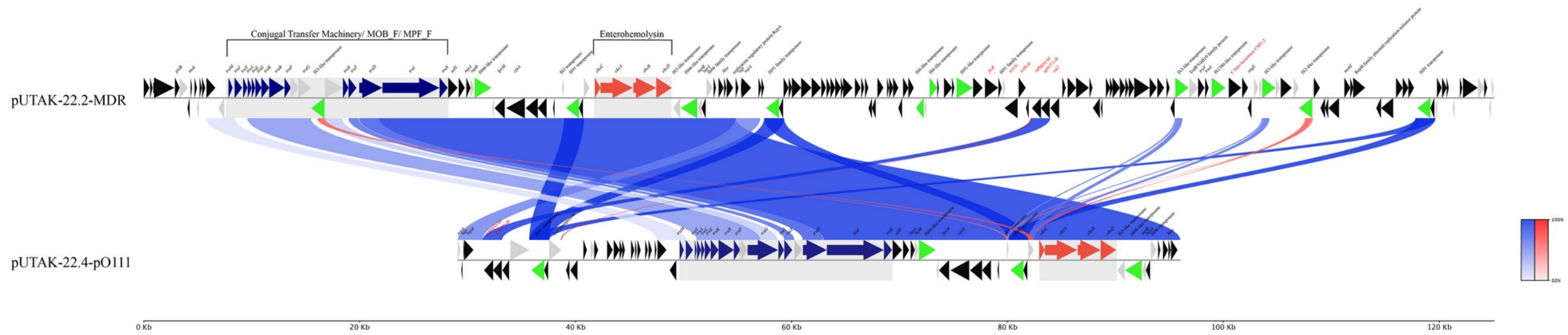

FIG S2. **Shared synteny between pUTAK-22.2-MDR and pUTAK-22.4-pO111.** Pairwise comparative alignment of pUTAK-22.2-MDR and pUTAK-22.4-pO111 showing extensive conservation across the IncF-like backbone, including replication/stability regions, the shared *ehxCABD* locus, and transfer-associated loci. Shaded links indicate nucleotide similarity between homologous regions. This comparison highlights the close structural relationship between the two plasmids while also showing accessory regions unique to each backbone, including multidrug-resistance cargo in pUTAK-22.2-MDR.

bioRxiv preprint doi: https://doi.org/10.64898/2026.09.16.752176; this version posted September 18, 2026. The copyright holder for this preprint (which was not certified by peer review) is the author/funder, who has granted bioRxiv a license to display the preprint in perpetuity. It is made available under a CC-BY 4.0 International license.

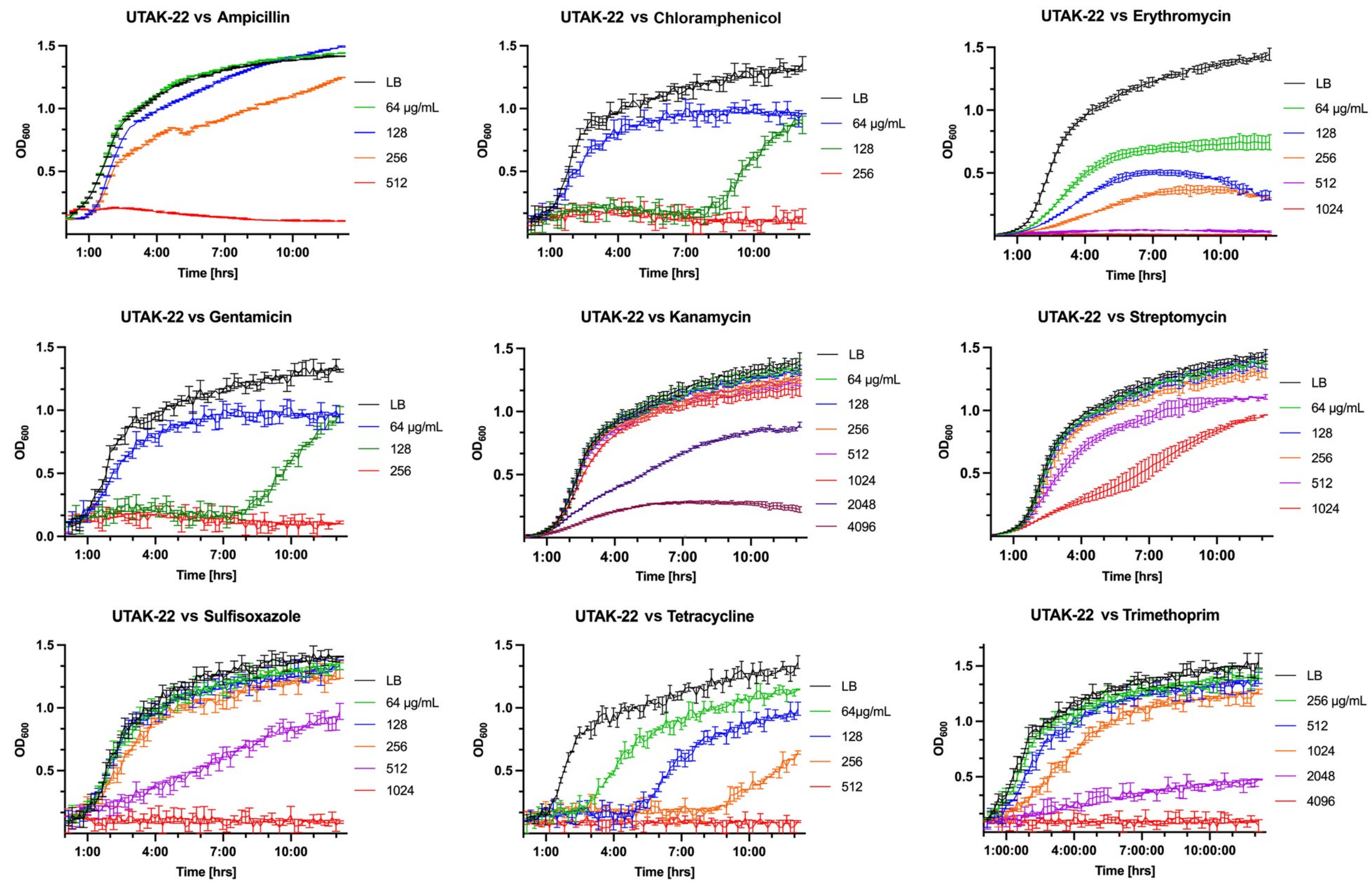

FIG S3. **Growth of UTAK-22 across multiple antibiotic conditions.** Growth curves of UTAK-22 in LB containing increasing concentrations of ampicillin (AMP), chloramphenicol (CHL), erythromycin (ERY), gentamicin (GEN), kanamycin (KAN), streptomycin (STR), sulfisoxazole (SFX), tetracycline (TET), and trimethoprim (TMP), monitored by OD_{600} over time relative to drug-free LB controls. These plots summarize the antibiotic-response phenotype of the native UTAK-22 plasmid background across multiple drug classes.

bioRxiv preprint doi: https://doi.org/10.64898/2026.09.16.752176; this version posted September 18, 2026. The copyright holder for this preprint (which was not certified by peer review) is the author/funder, who has granted bioRxiv a license to display the preprint in perpetuity. It is made available under a CC-BY 4.0 International license.

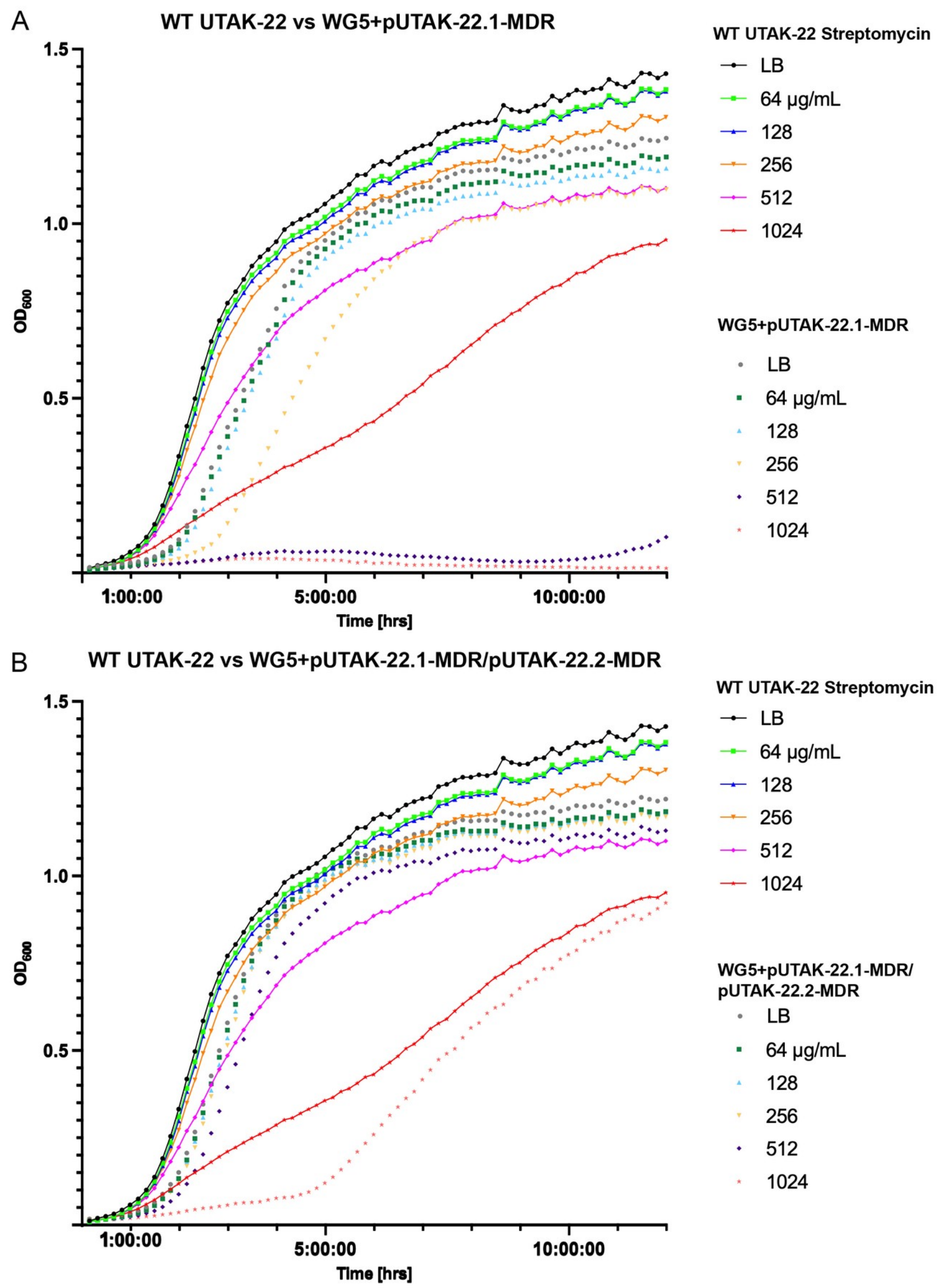

FIG S4. **Direct comparison of streptomycin growth kinetics between UTAK-22 and selected transconjugants**. Overlay growth curves comparing wild-type UTAK-22 with **(A)** WG5+pUTAK-22.1-MDR and **(B)** WG5 carrying the pUTAK-22.1-MDR/pUTAK-22.2-MDR plasmid combination across increasing streptomycin concentrations. Solid and dotted traces distinguish strain backgrounds as shown in the panel legends. The plots emphasize the higher streptomycin tolerance of the native UTAK-22 plasmid background and the partial restoration of high-dose growth in the co-transfer background relative to pUTAK-22.1-MDR alone.

bioRxiv preprint doi: https://doi.org/10.64898/2026.09.16.752176; this version posted September 18, 2026. The copyright holder for this preprint (which was not certified by peer review) is the author/funder, who has granted bioRxiv a license to display the preprint in perpetuity. It is made available under a CC-BY 4.0 International license.

Supplemental Tables

Table S1 **Strain-associated metadata and genome statistics**

Table S2 **Primers used in this study**

Table S3 **Achtman 7-gene MLST profiles and cgMLST allele calls (4,160 targets) for UTAK-22 and the 30 most closely related *E. coli* genomes**

Table S4 **Chromosome features**

Table S5 **Plasmid features**

Table S6 **Conjugation efficiency**

Supplemental Text

Evolutionary reconstruction of MDR cassette dynamics
